# Supplementary material for: Understanding the role of policy on inequalities in the intergenerational correlation in health and wages: Evidence from the UK from 1991–2017
Source: PLoS One. 2020 Jun 17;15(6):e0234737. doi: 10.1371/journal.pone.0234737 (PMC7299402; doi:10.1371/journal.pone.0234737)
Supplement: Attachment — Submitted filename: Points to Reviewer May 2020.docx [file pone.0234737.s002.docx]

I want to thank the editor and Reviewer for taking the time to review my initial revisions and make further suggestions. I hope that I have adequately addressed all your points. The original comments are in black and my reply is in red.

Reviewer 1, however, requests a four  additional clarifications. Although Reviewer 1 has "never seen such an analysis before" using the fixed effects model specification you employ (Point #1), I am familiar with such research in econometrics, specifically research on labor economics and health.  Reviewer 1's other concerns, especially Point #2, are valid; please explain why you employ a fixed-effects model over a difference-in-difference (DD) or a difference-in-difference-in-difference (DDD) approach to estimating the effects of the policy changes.  Please justify your use of estimating the average fixed effect in the population (before and after policy periods) compared to simply estimating the effect of the policy using DD or DDD approaches.

There are a number of reasons, why I chose to estimate a fixed effects model over an alternative specification such as a difference in difference or a difference in difference in difference. Because I am looking at multiple changes to the policy environment and I am not focusing on one specific element of policy, I did not think that a difference in difference approach would be appropriate. In the paper, I am interested in the whole policy environment and general attitudes by the government to health inequalities. A fixed effects approach which removes bias from time constant underlying factors is more suitable estimation approach in this scenario. A difference-in-difference model would be more appropriate if we were interested in evaluating one element of the health inequalities strategy or austerity to look at how that impacted on mobility. To employ a difference in difference approach, we would need a suitable control group where the underlying trends would be the same between the groups except for the policy. As the aim of this paper is to look at the policy environment, even though there are subtle differences between England, Scotland, and Wales as we are focused on central government ideology and policies towards inequalities; there are not enough differences for us to have a suitable control group in our data. Also, since we are interested in the policy environment using a difference in difference approach would make interpretation of the results challenging, as we wouldn’t know what was driving any conclusions and may inadvertently introduce more bias. With the fixed effects approach, we can draw some conclusions on the relationship between the policy environment and mobility, but we don’t necessarily know what is driving this relationship.

To justify this approach the following lines have been added to the text on page 8:

‘As we are not looking at specific elements of policy but the wider policy landscape, a more generalised model such as a fixed effect is more appropriate than a quasi-experimental model such as difference-in-difference. We do not have an obvious ‘treatment effect’ or control group. With this approach, we can draw some conclusions on the relationship between the policy environment and mobility, but we don’t necessarily know what is driving this relationship.’

Also, although you have edited your paper considerably, a number of minor typographical errors remain.  For example, in the second paragraph under the methodology section, "In2001" should be "In 2001," and "US" should be "USS".  Please edit your submission again to ensure that all acronyms, spacing, and other minor grammatical errors are fixed.

I am sorry for the remaining errors and have proofread the paper so hopefully I have caught all of them.

1- My major concern is related to the model used in the analysis. To me, a linear regression with fixed effects is used for panel data where 1) the explanatory variable is a random variable with a normal distribution and 2) where the unit of analysis is the individual. In your case, your explanatory variable is time expressed in year (without a normal distribution) and where the unit of analysis is time as well. Therefore I am questioning the validity of the model in this case. I have never seen such an analysis before so it would be good to have more explanation on this type of analysis.

See the comments above to the editor’s comments, regarding why a fixed effects model is appropriate. The aim of this research is to determine if the wider policy landscape and government views towards inequalities impact on mobility. We are not interested in evaluating a specific policy. If we find some changes in mobility over time after controlling for time constant factors (as we can do with the fixed effects model), this will motivate future work trying to identify specific policies that cause any observed change in mobility. For this type of analysis, a quasi-experimental approach such as difference-in-difference would be the best model choice.

2- Related to this point, I also don't understand how your model enables to attribute the changes observed to the change in policy landscape. To me the present analysis is purely descriptive and no inference can be made about any causal effect. I think that should be made clear.

Please see the points above, about the model choice. The aim of the model is to be descriptive to inform future analysis. If we find that there is some evidence that mobility changes over time then in future analysis, we can try to identify specific policies that cause this change.

To make this clearer I have added the following to the text on page 4 and 11:

Page 4: This is a descriptive analysis to understand how family mobility is associated with the policy environment.

Page 11: This paper is descriptive and does not specifically explore policy levers or mechanisms which may influence the role of the family on long term mobility. Future work should explore specific policies to identify causal relationships between policy and mobility.


3- I still don't understand how the cohorts are defined. From your table 1, it seems that the mean age for each of the 3 time periods are quite similar. If these cohorts are constituted with the same families, how come the age do not change over time? I must have misunderstood something in the construction of the cohorts, so maybe more explanation on this would be useful is the method section.

I have revised the text on the data section to make it clearer that we have different families. (See first paragraph on page 7:

‘In total we have data for 25 years on different families.’

I have revised the text at the start of the results section on the top of page 9 to hopefully make this clearer.

‘ Over the three time period, we want the mean characteristics of parents and children to be similar so that we know that any change in the correlation is attributable to other factors such as the policy environment rather than changing family composition.

4- I understand you are looking at the three outcomes separately to see if the correlation goes in the same direction over time. Given the interconnection between health and wages as you nicely described in the introduction, I would find it useful in the discussion to emphasise more on this aspect. The policy implications are a bit light at present.

The discussion has been revised to tie in better with the introduction. I have also expanded upon policy implications.
