# Supplementary material for: Understanding the role of policy on inequalities in the intergenerational correlation in health and wages: Evidence from the UK from 1991–2017
Source: PLoS One. 2020 Jun 17;15(6):e0234737. doi: 10.1371/journal.pone.0234737 (PMC7299402; doi:10.1371/journal.pone.0234737)
Supplement: Attachment — Submitted filename: Response to Reviewer Comments.docx [file pone.0234737.s001.docx]

Response to Reviewer Comments:

Firstly, I want to thank the editor and the reviewer for their helpful comments to improve the quality of the manuscript. I hope that all of the comments have been addressed. Please note the comment is in black and my reply is in red.

**Comments from the Editor:**

First, and importantly, you should define SAH in the abstract and earlier in the paper.

SAH has now been defined in the abstract and in first use in the paper in the first paragraph on page 4.

Along these lines, please change the acronym for the Understanding Society Survey (US) to USS to prevent any confusion with the United States (US) in your discussion.

This acronym has been changed throughout the text.

Second, are the British Household Panel Survey (BHPS) and USS (see above) data based on random samples of households?  If so, you should state that this fact.

The two studies: BHPS and USS are drawn from a random sample of households. A two stage sampling procedure was used. Primary selection was based upon postcode which were then grouped into geographical strata to ensure a nationally representative selection of households.

The text above has been incorporated into the description of the data on the bottom of page 6 and the reference relating to sample selection:

Al Baghal, T., A. Jaeckle, et al. (2015). Understanding Society -The UK Household Longitudinal Study: Innovation Panel, Waves 1-8, User Manual. Colchester, ISER University of Essex.

Has been added to the reference list [29].

Third, are wages adjusted for inflation across time periods?  If not, please do so.  In the manuscript, you should also state whether wages have been adjusted for inflation.

The wages have been adjusted for inflation, the base year was 2016. This is now mentioned in the text on the middle of page 7.

Lastly, please edit your manuscript for minor grammatical problems.  For instance, you should change "23,764 observation" to "23,764 observations" near the bottom of page 6.  Similarly, change "Our results show that for the population on average," to "Our results show that for the population, on average," on page 9.

The document has been proofread throughout and the specific errors mentioned above have been revised.

**Reviewer 1:**

- It would be good to know what is considered a high/low intergenerational correlation. Are there any standards in the literature? One can look at the sign of the correlation, but it would be good if for example 0.25 is considered as a high correlation in this domain.

This is a complicated question. There is a well-established literature investigating the intergenerational correlation in wages/earnings The correlation is very country specific (see for example Blanding 2011 [15]) it ranges from 0.15 in Norway to 0.45 in France with an overall mean of approximately 0.22. Where there is significantly less literature on the intergenerational correlation in health. Looking at the literature cited in the paper, the intergenerational correlation in health ranges from 0.16 (mental health) to 0.20-0.25 (self-assessed health).

Given this, the correlation from Blanden has been added to a discussion of the literature on the intergenerational correlation in income/earnings (see bottom of page 2) to put the correlation in context.

The following text has also been added to the introduction (see middle of page 3):

‘Thus, given the existing literature the mean correlation in wages/earnings is approximately 0.20 which is very similar to the mean correlation in health. However, it is important to note that there is a lot of variation across this correlation and some of this variation is country dependant…‘

Reference: Blanden J. Cross‐country rankings in intergenerational mobility: a comparison of approaches from economics and sociology. Journal of Economic Surveys. 2013 Feb;27(1):38-73. (Reference number 15).

- It is unclear from the introduction which outcomes are going to be looked at and how. The novelty of this study is that it looks at the "intergenerational correlation in health and wages". From this statement, it seems that the correlation studies will be between childhood health and adult wages, which is not the case. Instead, health and wages are two separate outcomes in the analysis. In other words, the correlation is only between generations rather than outcomes. It is a bit confusing since a substantial paragraph is dedicated to the Grossman model emphasising the correlation between health and wages. Maybe you should be a bit clearer from the beginning on what outcome you are going to look at and how you will assess correlation.

Thank you for pointing this out. I found it a difficult concept to try and communicate and I don’t think I had got the point across clearly.

The abstract, introduction, and theoretical framework section have been revised to make it clearer that what the paper is trying to do is look at multiple outcomes across generations rather than the correlation between health and wages. See for example the final paragraph on page 3:

‘Estimating correlations in multiple outcomes, such health and earnings between generations are an important component of understanding mobility. Mobility is multidimensional and should not be limited to a single domain such as earnings. Thus, in order to create effective policy to ensure equity of opportunity for all it is necessary to understand how policy may impact on multiple dimensions of mobility such as health and wages. For the development of future policy, it is essential to understand how the strength of the correlation across these multiple outcomes is changing over time. As different dimensions may require a bespoke policy solution if the strength of the correlations in outcomes are moving in opposite directions. If government policy can influence the strength of the intergenerational correlation across multiple dimensions of mobility then this would be one key mechanism to influence mobility in the medium/long term.’

The research questions have also been revised to try and make this clearer as well see bottom of page 6:

1) Did a systematic focus on reducing child poverty and health inequalities over the period 1999-2010 impact on the strength of the relationship between generations in both health and wages? Was the impact same or different for the two outcomes of health and wages?
2) Is there a differential impact of policy focus on the strength of the relationship between generations in health and wages across the socioeconomic spectrum as measured by parental educational attainment, social class, and for single parent families compared with two parent families?

Data and methods:

- More information should be given about the surveys. Are they conducted yearly? How many data points do you have?

Both surveys are conducted annually. We have annual data over the period 1991-2016 or 25 data points.

This information has been added to the text in the penultimate paragraph on page 6:

All household members 16 years old and older annually in both surveys

And in the final paragraph on page 6:

In total we have data for 25 years.

- How are generations defined? In the first survey, you have one cohort of individuals divided into two policy periods. Given that the same individuals (two generations) will be use for the analysis of period 1991-1999 and 1999-2010, how do you account for the fact that the correlation in each outcome in the second period will be largely influenced by the correlation in the first period?. This is even more problematic since the the last period 2010-2017 is drawn from a separate sample.

Generations are determined by parents and their adult children (aged 16 and over) from matching on parental identification numbers in the sample. As mentioned in the paper on the top of page 6, there is continuity of respondents between the BHPS and USS (6700 respondent from the BHPS joined the USS) so that the last period (2010-2017) is not a completely different sample. To remove individual/family effects on the correlations these are the means across the sample (this is explained in more detail below to some of the other comments from the Reviewer). As the correlation is the population mean, an individual’s family correlation from last year should not have a significant influence on the correlation in the current period. Because we use population level correlations, as long as the sample is relatively representative of the population it should not matter how many waves an individual appears in.

To address this question the following has been added to the text:

‘In the analysis, we are interested in the relationship between generations in both health and wages. We define generations as parents and their young adult children (aged 16+). Parents and children are identified in the dataset using the study id of the mother and/or father. This id is available in the data for all children of the parents irrespective of the child’s age.’

- The correlation in each outcome has potentially lots of determinants other than policy landscape. Using the "mean correlation for the sample for each wave" is confusing: how does this remove the effect of genetics and other key determinants?? It seems that the econometric model lacks theoretical grounds.

As clarified for the previous comment regarding the unit of analysis. We take the mean correlation for the sample or sub-sample for the outcome variable. This is the mean for each year so we can look at variation in the mean over the 25 years of data. As we look at the mean across the sample, we remove family level effects from the correlation. The theoretical grounds for this is that by summing across families we remove the individual family genetic component from the outcome variables as well as the impact of transitory fluctuations at the family level.

In terms of other factors which are likely to impact on each outcome, that is part of the reason why we employ a fixed effect model, if there are other time constant unobservable factors which are likely to influence this relationship (which is likely to be the case) a fixed effects approach will remove the bias on our explanatory variables from this (see also the comment to the next point which expands upon this).

The final paragraph on page 7, has been revised to make this clearer.

Why do you use fixed effects? More explanation should be provided.

If there are unobserved time constant factors impacting on the correlation between generations in health and wages using a fixed effects approach will remove this bias. As mentioned on the top of page 9, we test this assumption using the Hausman test to determine if fixed effects is appropriate or if a random effects specification which only accounts for individual heterogeneity but does not reduce any bias from omitted variable bias (time constant factors being correlated with the explanatory variables). The Hausman test shows that a fixed effect model is the more efficient estimator.

To clarify, the use of fixed effects the following sentence has been added to the text on the bottom of page 8.

‘Fixed effects are used to reduce bias from time constant factors related to the wider landscape impacting on the correlation between generations.’

- How correlation is measured? At the individual level by pairing each child with parent? This is critically important but not clear enough.

The correlation is measured at the population level by taking the mean of all the individual family correlations in the outcome variables. This is clarified on page 7-8.

- The unit of analysis of the econometric model is unclear, is it at the individual level? Or is it average correlation? This is unclear. Do you use any clustering to account for family effect?

The unit of analysis is the mean correlation of the all families in the sample or sub-group. Because we are not estimating correlations for individual families the analysis does not account for any clustering at the family level. The following sentence clarifying this has been added to the text on the middle of page 7:

‘Thus, we are not estimating correlations for individual parent and children but the average for the sample and average correlations for the socioeconomic characteristics of interest.’

And this is also clarified in the revised outcome variables on the top of page 8 as well as the revised explanation of the model on page 8.

- How do you take inflation into account?

Please see comment above to the editor on how inflation is accounted for.

Results and discussion:

- Be careful you have two figure 1.

I have checked the figures to ensure that there is only one figure 1.

- you should emphasise in your limitations that measuring health by SAH is not great, since it is subject to lots of biases. Although, it is even more problematic since this bias might not go in the same direction by SES, which can cause problems for the sub-group analysis. Could you use presence chronic disease as a measure of health for example?

SAH is chosen to be comparable with the literature (see for example references [17,19].) If there is a bias in SAH by SES as long as it was going in the same direction for parents and children then it should not bias our findings.

There are not consistent measures of chronic disease in the dataset which can be used over the 25 year period.

To address this comment, we have added the following sentences to the penultimate paragraph on page 11:

‘There may also be measurement error with our outcome variables as they are self-reported. Even if this measurement error is systematic by our SES indicators, if it moves in the same direction for parents and their children this should minimise the bias in our findings.’

- Figures 2a,b and c are very difficult to read.. might be useful to present this in another way.

These figures have now been separated by outcome measure for each of the three socioeconomic variables. (See Figures 2A, 2B, 2C, 3A, 3B, 3C, and 4A, 4B, 4C). The text related to the Figures has been revised to reflect this change.

- "The findings suggests that the impact of government policy related to reducing inequalities on
the family unit is lower than the impact on individuals." Where is the result supporting this statement?

We have revised the text on the top of page 11 to make this clearer, that the evidence at the individual level found a large impact of the policy period on outcomes such as infant mortality and geographical health inequalities. Whereas we found less evidence that policy period changed the influence of parents on their young adult children’s outcomes.

‘Results from studies at the individual level such as [23,24] found large impact of the health inequality strategy period on geographical health inequalities and infant mortality respectively, where we found less evidence that policy period changed the influence of parents on their young adult children’s outcomes.’

- the statement "Our findings provide some evidence that the correlation in health and wages does evolve over
time and may be responsive to changes in policy focus especially for those at the highest and
lowest end of the socioeconomic spectrum." does not seem to be fair considering that poor correlation has been found overall.

This statement has been revised on page 11 (see below). However, we do find some evidence that at the top and bottom end of the socioeconomic spectrum there is some responsiveness of the influence of parents on their young adult children’s health and wages so I think this statement is fair.

Our findings provide some evidence that the influence of parents on their young adult’s health and wages does change over time and may be responsive to changes in policy focus especially for those at the highest and lowest end of the socioeconomic spectrum.
